# Modifying landing mat properties elicits different lower limb biomechanical responses in gymnasts and non-gymnasts

**DOI:** 10.1038/s41598-025-26522-y

**Published:** 2025-11-27

**Authors:** Pavel Brtva, Lucia Bizovská, Bethany Canty, Jiri Skypala, Gareth Irwin, Roman Farana

**Affiliations:** 1 Department of Human Movement Studies, University of Ostrava, 701 03 Moravska Ostrava, Czech Republic; 2 Department of Natural Sciences in Kinanthropology, Palacký University Olomouc, Olomouc, Czech Republic; 3 Research Institute for Sport and Exercise, University of Canberra, Canberra, Australia; 4 Cardiff School of Sport and Health Sciences, Cardiff Metropolitan University, Cardiff, UK

**Keywords:** Landing mats, Testing, Lower-limb joints, Kinetics, Kinematics, Engineering, Physiology

## Abstract

This study examined vertical ground reaction force (VGRF) and lower-limb kinematics during drop landings on different gymnastics mat types. Responses were compared between skilled female gymnasts (n = 11) and untrained young females (n = 11). Participants completed six standardized drop landings on a FIG-certified mat, a gymnastics carpet, and three custom-designed mats (TYPE 1–3) varying in layer number and material properties. Kinetic and kinematic data were collected for each trial. A two-way mixed ANOVA examined the effects of mat type and group. Both mat characteristics and participant background significantly influenced landing biomechanics. This included VGRF_max_, time to VGRF_max_, peak ankle and knee joint angles, and peak knee angular velocity. The FIG-certified mat produced the lowest peak forces and the longest time to peak. The carpet surface generated the highest peaks and shortest times. Softer mid-layers, as in TYPE 2, were associated with more favourable force profiles. Stiffer constructions transmitted higher loads. Gymnasts exhibited consistent plantarflexed initial contact, reduced knee flexion, and lower knee range of motion across surfaces. This reflects a joint-stiffening strategy likely developed through training. Non-gymnasts adopted deeper, more compliant landings and adjusted more to softer mats. These findings highlight the role of mat structure and user experience in shaping landing biomechanics. The results underscore the importance of biomechanical analysis in landing mat development to enhance safety across skill levels.

## Introduction

In general, performing gymnastics skills requires explosive, balance, and artistic skills, including movements with a high level of intensity and difficulty^1^. Female gymnastics skills are performed on various types of apparatuses (i.e., vault, uneven bars, balance beam, floor), where skills and routines are concluded with a dismount and landing. In gymnastics, landing represents a fundamental skill, which is practiced continually throughout all levels^2^. Previous studies highlighted that competitive gymnasts aged from 7 to 18 years are exposed to high training loads of up to 21–37 h per week, for 11–12 months per year^3–5^. Moreover, a review study by Gittoes and Irwin^6^ showed that during a single training week, female gymnasts performed more than 200 landings. Concerning the age of female gymnasts, it must be considered that immature musculoskeletal systems, growing cartilage, articular cartilage, and bones are more prone to injury risks^7^. The results of epidemiological studies show that in artistic gymnastics, the lower limbs, specifically, the knee and ankle joints, represent the most injured part of the body^8^^,^^9^. These injuries commonly happen in the landing phase after multiple rotations, when the knee and ankle joints are subjected to high mechanical loads^6,10^. Moreover, previous findings suggest 49–52% of these injuries occur during the landing phase and dismounts^11^^,^^12^.

The main goal of gymnastics landing is to safely reduce all body momenta to zero with simultaneous placement of both feet whilst adhering to the expectations stated in the Code of Points^13^. Landing success relies on a gymnast’s physical preparation to withstand landing forces and motor control to execute the skill, with both factors essential for safe and effective performance. Physical preparation refers to a gymnast’s capacity to withstand the forces encountered during landing, and motor control is the gymnast’s ability to regulate and execute the skill they perform^14^. Therefore, controlling multiple joints to manage the substantial internal and external forces encountered during landing poses a considerable challenge to the neuromuscular system both before and during contact with the landing surface^15^. An important issue for research is the landing constraints, which are influenced by landing surfaces and their role in the Gymnastics Code of Points (Federation Internationale de Gymnastique, FIG). Landing criteria for judging in gymnastics dictate that gymnasts must land with a single placement of the feet (no additional steps) with the centre of mass over the base of support. Any steps or unsteadiness, excessive arm swinging, or loss of balance can result in a score deduction between 0.1 and 0.5 points^13^. Therefore, a successful landing from dismount can make a difference between winning or losing^16^.

To help reduce impact forces, a variety of landing surfaces have been developed, which are commonly assigned to one of two groups: point-elastic surfaces that distribute forces over a small area, and area-elastic surfaces that react to a local force by deforming over a relatively large area^17^. The stiffness of the landing surface is a basic parameter that distinguishes different types of landing surfaces and has previously been highlighted, as well as different responses of gymnasts’ musculoskeletal systems^18–21^. In recent years, there have been changes in the rules for the construction of impact surfaces, which must comply with the FIG regulations. For this reason, landing mats are made from different materials (e.g., different density foams, polyethylene, rubber) and structures (i.e., number of layers,usually top, mid, and bottom) and are constantly being developed to increase the safety of athletes. Testing of landing surfaces according to FIG rules (FIG apparatus norms) and standardization procedure is carried out using a predefined impactor (20 kg ± 0.2 kg,Ø 10 cm ± 0.5 cm) that impacts with an impact velocity of 3.96 m/s (corresponding to a height of 0.8 m). Moreover, the results of this testing protocol must not exceed the limits of the selected parameters (deflection, height of rebound, and resultant force maximum). Findings of previous studies state that the issue of testing different types of mats does not consider the biomechanical loading of the athletes’ musculoskeletal system and the repetitive mechanical loading of the lower limbs^22,23^. Additionally, a preliminary study by Brtva et al.^24^ indicated differences in reaction forces between landing mats usually used during gymnastics training and competition with the same FIG certification, but with a different structure. Previous research findings highlight that the development of landing mats needs to respect the biomechanical responses of the musculoskeletal system of athletes using biomechanical analysis. Therefore, the purpose of this study was to determine how the different structures of landing mats differ in the vertical component of the ground reaction force and lower limb joints kinematics during landing. Three different gymnastics mats under development and a standard gymnastics carpet were compared to the FIG-certified mat. Due to use of the three vs. four-layer mats with different foam used, it was hypothesized that during landing on these mats: (1) the VGRF maximum would significantly differ between the mat types, with lower peak forces expected on mats featuring softer or more compliant mid-layer structures compared to stiffer mats and the gymnastics carpet,and (2) kinematics of the lower limb joints will differ between gymnasts and non-gymnasts, as well as across mat types, reflecting both surface compliance and movement strategy adaptations. This research may provide important information to improve safety not only for gymnasts but also for non-gymnasts, as landing mats are widely used in school physical education. It also highlights the importance of incorporating biomechanical analysis into the testing and development of landing mats.

## Methods

### Participants

Purposeful sampling was used to select eleven young, active female artistic gymnasts (GYM) from the Czech Republic, each with over 5 years of systematic training and competitive experience (age: 10.7 ± 1.1 years; height: 144.3 ± 6.2 cm; mass: 37.2 ± 8.2 kg). The control group (CON) consisted of eleven age-matched females without gymnastics experience (age: 9.6 ± 1.8 years; height: 135.9 ± 15.2 cm; mass: 32.8 ± 11.7 kg). All participants were free from lower extremity injury, and informed consent was obtained from each gymnast and their parents.

### Protocol

**Fig. 1:**
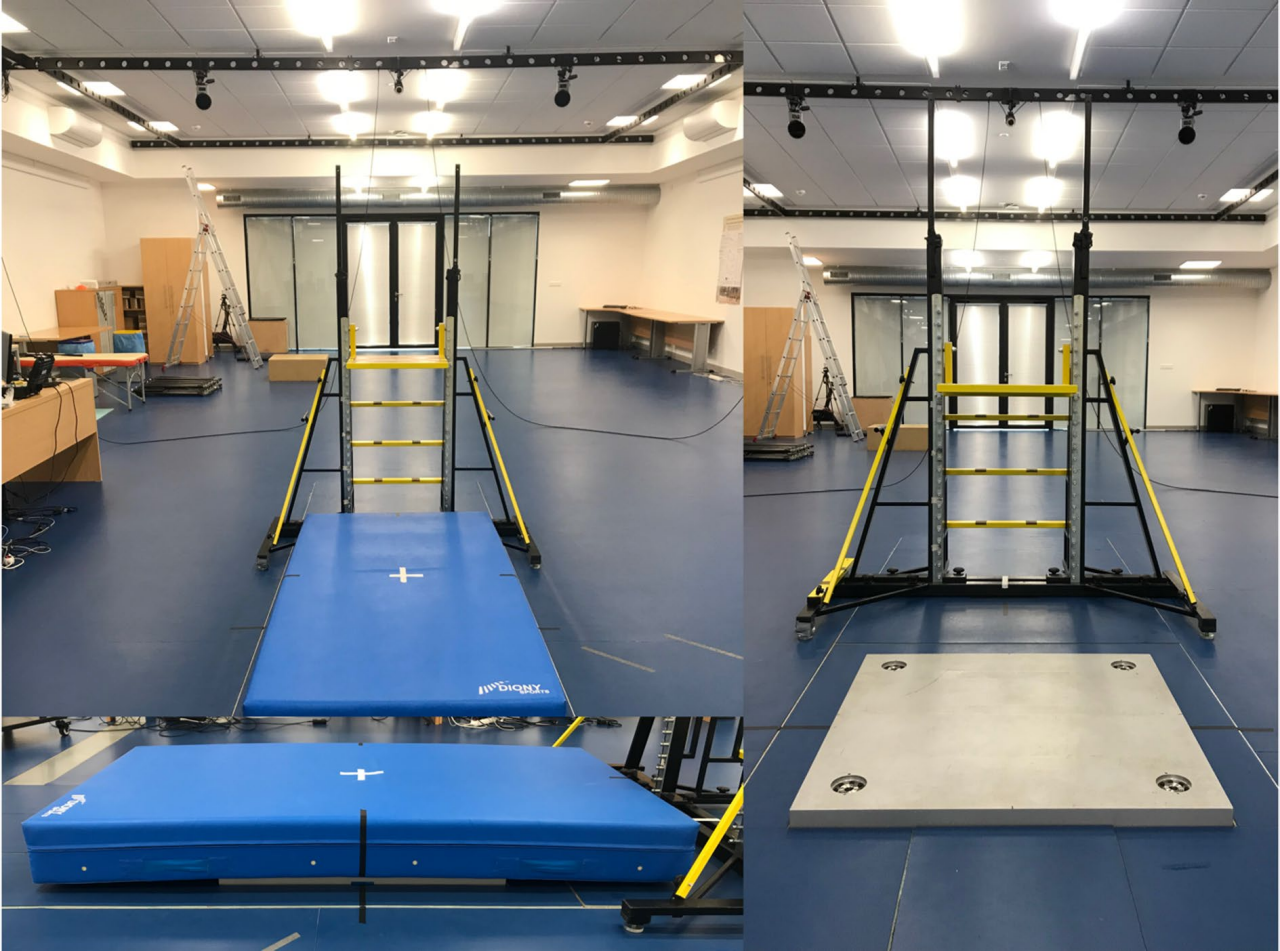
Step-off platform and force plate set-up.

The study design, including the experimental protocol and measurement methods, was approved by the Ethics and Research Committee of the University of Ostrava (OU-27383/45-2023), and all procedures were carried out in accordance with the relevant guidelines and regulations, including the Declaration of Helsinki. The main measurement took place in a biomechanical laboratory at a Human Motion Diagnostic Centre at the University of Ostrava. After a self-selected warm-up and practice, each gymnast performed 6 trials of the drop-landing task onto each different landing mats: gymnastics carpet, TYPE 1, TYPE 2, TYPE 3, and FIG-certified mat. Participants were instructed to perform a competition landing style in barefoot conditions. The drop landings were performed stepping off from a platform (without pushing or hopping with their preferred lower limb) of an approximately 0.80 m high to replicate typical landing velocities experienced by gymnasts on apparatus such as the floor and balance beam (McNitt-Gray 1991) onto a force plate covered with gymnastics mats (Fig. 1). All trials were performed in a random order of the used mats.

### Landing mats structure

**Fig. 2:**
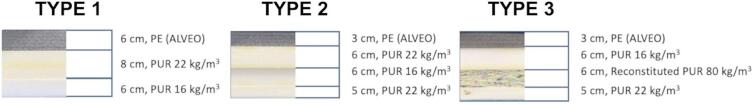
Schematic diagram of different landing mats structure.

Four different types of mats were used in the current study: a FIG certified mat and three prototype mats, referred to as TYPE 1, TYPE 2, and TYPE 3 (see Fig. 2). All mats measured 2000×1000×20 cm and were covered by synthetic leather. They were constructed from polyethylene foam (PE), polyurethane foam (PUR), and reconstituted PUR at varying densities (kg/m^3^—higher values indicate stiffer layers). The key differences among the mats are as follows: TYPE 1 (three-layer) comprised a 6 cm thick stiff PE top layer, an 8 cm PUR (22 kg/m^3^) middle layer, and a 6 cm PUR (16 kg/m^3^) softer bottom layer; TYPE 2 (four-layer) consisted of a 3 cm thick stiff PE top layer, a 6 cm PUR (22 kg/m^3^) second layer, a 6 cm PUR (16 kg/m^3^) soft mid layer, and a 5 cm PUR (22 kg/m^3^) bottom layer; TYPE 3 (four-layer) included a 3 cm thick stiff PE top layer, a 6 cm PUR (16 kg/m^3^) second layer, an 8 cm reconstituted PUR (80 kg/m^3^) stiff mid layer, and a 5 cm PUR (22 kg/m^3^) bottom layer. To allow for better comparison regarding ground reaction force reduction and changes in landing technique, an additional condition involved landing on a 3 cm thick gymnastics carpet (PE).

### Experimental set-up

One force plate (900 × 900mm, Kistler, 9287CCAQ02, Switzerland) was used to determine ground reaction force data at a sampling rate of 2160 Hz. To obtain valid ground reaction data, the top of the force plate was raised by 0.04 m so that the mats placed on the platform did not touch the surrounding ground^25^, see Fig. 1.

**Fig. 3:**
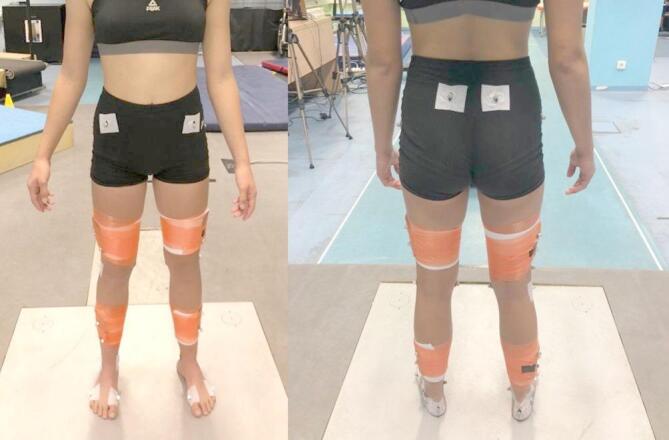
Marker placement on gymnasts’ lower extremities and pelvis.

To collect the kinematic data a motion-capture system (Qualisys, Inc., Gothenburg, Sweden) consisting of 18 infrared cameras was used at a sampling rate of 240 Hz. Data from the force plate and the cameras were synchronized and collected simultaneously. Before measurement, a global coordinate system was calibrated with a wand calibration kit based on Qualisys’s recommendation (Qualisys, Inc., Gothenburg, Sweden). Based on HAS-Motion (Kingston, Ontario, Canada) recommendations, 40 retroreflective markers (diameter of 12 mm) and clusters were attached to the gymnasts’ lower limbs and pelvis (Fig. 3). Markers were bilaterally placed on each participant at the following anatomical locations: pelvis bilaterally and on the anterior and posterior superior iliac spines, right and left greater trochanters of the femur, the medial and lateral femoral condyles, and the medial and lateral malleoli, head of the first and fifth metatarsal heads along with three markers placed on the heel. Four clusters containing markers were also placed bilaterally on the thigh and shank.

### Data analysis

Raw coordinate data were processed using Visual 3D software (version 4; HAS-Motion, Kingston, Ontario, Canada). The local coordinate systems were defined using a static calibration trial in the stand position. All lower extremity segments were modelled as truncated cones. Based on the kinetic data, the contact phase was identified when the vertical component of the GRF exceeded 20 N. All analyses focused on the first phase of landing (0–300 ms). The coordinate data were low-pass filtered using a fourth-order Butterworth filter with a 12 Hz cut-off frequency, and force data were filtered using a fourth-order Butterworth filter with a 50 Hz cut-off frequency, customized through residual analysis^26^. The dependent variables selected for analysis were maximum vertical ground reaction force (VGRFmax), time to peak VGRFmax, knee and ankle joint flexion/extension, as well as the range of motion in the sagittal plane, and knee and ankle joint angular velocities. The absolute force data were subsequently normalized to body-weight units, allowing for a more objective comparison of differences.

### Statistical analysis

A two-way mixed analysis of variance was conducted with mat type (carpet, TYPE 1, TYPE 2, TYPE 3, FIG) as the within-subjects factor and group (GYM vs. CON) as the between-subjects factor. Pairwise comparison was performed by the Bonferroni post-hoc test on a significance level of 0.05. Statistical analysis was performed in software IBM SPSS (v. 26, IBM, NY, USA).

## Results

Descriptive statistics with means and standard deviations for all conditions are presented in Table 1.

**Table 1:** Descriptive statistics of kinetic and kinematic measures mean (SD).

Variable	CARPET		TYPE 1		TYPE 2		TYPE 3		FIG	
	GYM	CON	GYM	CON	GYM	CON	GYM	CON	GYM	CON
θAnkle_max_ (°)	17.42 (4.82)	23.76 (6.19)	12.89 (6.56)	19.22 (7.11)	12.99 (6.82)	18.64 (5.72)	12.32 (6.35)	18.28 (7.86)	10.15 (6.03)	16.40 (5.82)
θKnee_max_(°)	− 97.80 (8.41)	− 118.60 (21.61)	− 89.94 (12.36)	− 110.84 (20.95)	− 89.65 (13.12)	− 112.94 (22.73)	− 87.68 (11.85)	− 109.36 (24.22)	− 82.95 (13.80)	− 109.26 (24.50)
ωAnkle_max_(°/s)	945.35 (152.73)	888.57 (245.09)	960.70 (264.71)	934.11 (160.91)	904.35 (273.13)	860.28 (256.46)	916.65 (242.72)	932.38 (183.67)	875.59 (235.75)	916.95 (233.89)
ωKnee_max_(°/s)	− 1179.39 (102.63)	− 1210.23 (186.32)	− 909.29 (113.14)	− 1021.69 (215.83)	− 890.33 (96.75)	− 997.42 (209.85)	− 880.02 (121.23)	− 963.53 (175.93)	− 792.99 (99.75)	− 921.74 (207.19)
θAnkle_ROM_ (°)	48.11 (6.35)	49.31 (10.61)	44.43 (8.90)	50.77 (8.15)	43.61 (9.95)	45.57 (10.69)	43.21 (8.40)	48.47 (9.40)	42.31 (8.14)	47.03 (8.28)
θKnee_ROM_ (°)	84.84 (9.14)	100.15 (21.36)	78.92 (9.57)	96.27 (20.85)	79.37 (10.71)	97.61 (21.86)	76.85 (10.93)	94.79 (23.61)	73.56 (11.22)	94.28 (23.38)
VGRF_max_ (BW)	11.49 (2.12)	11.05 (2.91)	7.46 (0.48)	7.40 (1.11)	7.31 (0.49)	7.00 (0.75)	7.42 (0.47)	7.12 (0.94)	7.17 (0.42)	6.91 (0.73)
Time to Peak VGRF_max_ (ms)	30.74 (2.13)	29.04 (7.43)	40.34 (5.14)	36.05 (7.62)	44.76 (4.77)	38.32 (8.41)	43.50 (4.66)	39.58 (7.66)	49.18 (3.24)	42.36 (8.22)

BW, body-weight; CON, non-gymnasts; GYM, gymnasts; max, maximum; °, degree; θ, joint angle (°); ω, angular velocity (°/s).

The results of the two-way mixed ANOVA are presented in Table 2. A significant main effect of mat type was found for VGRF_max_ (*p* < 0.001). Post hoc analyses indicated that the carpet surface produced significantly higher peak forces than all tested mats (*p* < 0.001), with the FIG-certified mat yielding the lowest values (GYM: 7.17 ± 0.42 BW; CON: 6.91 ± 0.73 BW) and the carpet the highest (GYM: 11.49 ± 2.12 BW; CON: 11.05 ± 2.91 BW). No significant differences were observed between the FIG mat and TYPE 2 or TYPE 3 mats (*p* = 1.000). Likewise, comparisons between TYPE 2 and TYPE 3 (*p* = 1.000), TYPE 1 and TYPE 3 (*p* = 1.000), and TYPE 1 and TYPE 2 (*p* = 0.074) were not statistically significant.

**Table 2:** Results of Two-way mixed ANOVA.

Dependent Variable	Mat (F)	Mat (p)	Mat (η^2^)	Group (F)	Group (p)	Group (η^2^)	Interaction (F)	Interaction (p)	Interaction (η^2^)
VGRF_max_ (BW)	72.898	< 0.001*	0.785	0.459	0.506	0.022	0.106	0.778	0.005
Time to Peak VGRF_max_ (°/s)	134.566	< 0.001*	0.871	3.373	0.081	0.144	4.098	0.007*	0.170
θAnkle_max_ (°)	23.780	< 0.001*	0.543	5.791	0.026*	0.225	0.076	0.989	0.004
θKnee_max_(°)	8.460	< 0.001*	0.297	9.658	0.006*	0.326	0.545	0.595	0.027
ωAnkle_max_(°/s)	0.966	0.419	0.046	0.027	0.872	0.001	0.647	0.597	0.031
ωKnee_max_(°/s)	158.374	< 0.001*	0.888	1.976	0.175	0.090	3.481	0.011*	0.148
Ankle ROM (°)	2.403	0.102	0.107	1.482	0.238	0.069	0.882	0.423	0.042
Knee ROM (°)	4.848	0.009*	0.195	6.704	0.018*	0.251	0.440	0.679	0.022

BW, body-weight; *, *p* < 0.05; VGRF, vertical ground reaction force; max, maximum; θ, joint angle (°); ω, angular velocity (°/s); η^2^—partial eta-squared.

No significant main effect of group was found (*p* = 0.506), and there was no significant mat × group interaction (*p* = 0.778).

For the time to peak VGRF_max_, a significant main effect of mat was observed (*p* < 0.001), with the longest times on the FIG mat (GYM: 49.18 ± 3.24 ms; CON: 42.36 ± 8.22 ms) and the shortest on the carpet (GYM: 30.74 ± 2.13 ms; CON: 29.04 ± 7.43 ms). A significant mat × group interaction was found (*p* = 0.007), whereas the main effect of group did not reach significance (*p* = 0.081). Pairwise comparisons for time to peak VGRF_max_ indicated significant differences between carpet and across all tested mats (*p* < 0.001), with the only exception being the comparison between TYPE 3 and TYPE 4, which did not differ significantly (*p* = 1.000).

In the sagittal plane, a significant main effect of mat type was found for both θAnkle_max_ (*p* < 0.001) and θKnee_max_ (*p* < 0.001). Pairwise comparisons for θAnkle_max_ revealed that the carpet surface differed significantly from all other mats, indicating distinct landing characteristics. The FIG mat also showed significant differences compared with TYPE 1 (*p* = 0.029) and TYPE 3 (*p* = 0.019), whereas no significant differences were observed among TYPE 1, TYPE 2, and TYPE 3 (*p* = 1.000). Additionally, the FIG-approved mat did not differ significantly from TYPE 2 (*p* = 0.099). For the θKnee_max_ pairwise comparisons indicated that the carpet surface differed significantly from TYPE 1 (*p* = 0.040), TYPE 3 (*p* = 0.009), and the FIG mat (*p* = 0.013). TYPE 1 and TYPE 3 showed no significant differences with any other tested mats, while TYPE 2 differed significantly only from the FIG mat (*p* = 0.013).

Furthermore, a significant main effect of group was found for θAnkle_max_ (*p* = 0.026) and θKnee_max_ (*p* = 0.006). Gymnasts exhibited greater plantarflexion than controls across all mats, while controls showed significantly greater knee flexion. No significant interactions were observed for joint angles.

For the ankle angular velocity (ωAnkle_max_), no significant main effects or interactions were found. However, a significant main effect of mat was identified for knee angular velocity (ωKnee_max_) (*p* < 0.001), with a significant mat × group interaction (*p* = 0.011). Pairwise comparisons for ωKnee_max_ showed that the carpet surface differed significantly from all other mats (*p* < 0.001). The FIG mat also demonstrated significant differences compared with TYPE 1 and TYPE 2 (both *p* < 0.001) and with TYPE 3 (*p* = 0.001).

Ankle range of motion (θAnkle_ROM_) did not show significant effects. However, for θKnee_ROM_, both a significant main effect of mat (*p* = 0.009) and of group (*p* = 0.018) were found, with CON showing consistently larger ranges (e.g., FIG: 94.28 ± 23.38°) compared to GYM (e.g., FIG: 73.56 ± 11.22°).

## Discussion

The present study aimed to examine vertical ground reaction force and knee and ankle joint kinematics during landings on different types of landing mats, while also determining potential differences between skilled and unskilled young females. Our results demonstrate that both mat properties and participant background significantly influence landing mechanics, including VGRFmax, time to VGRFmax, peak ankle joint angle, peak knee joint angle, and peak knee angular velocity.

One of the most striking findings was the strong main effect of mat type on VGRFmax and time to peak VGRFmax. The FIG mat produced the lowest VGRFmax and the longest time to peak, consistent with its purpose-built shock-absorbing PUR foam. In contrast, the carpet surface generated the highest peak forces and the shortest time to peak, reflecting its low compliance and limited energy dissipation. Among the custom mat designs, TYPE 1 (the stiffest mat, with three layers and a stiff top PE layer) produced the second-highest peak forces, while TYPE 2 (featuring a softer PUR mid-layer) yielded force profiles more similar to FIG standards. TYPE 3, with a stiffer reconstituted PUR mid-layer, exhibited intermediate behavior. These distinctions underscore the influence of both the number of layers and the mechanical properties of the mid-layer material on landing load transmission. These results suggest that different mat structures substantially influence impact forces during landing, which aligns with previous research emphasizing the role of landing mechanics in injury prevention^23,24^. In the case of the TYPE 2, the mat structure produced results most similar to those of the FIG-certified mat. A combination of softer layers was placed in the middle to enhance the absorption of reaction forces, while stiffer layers were used in the top layer to help stabilize the ankle joint, as suggested previously by^4^, seems to be a key factor. Time to peak VGRFmax followed a similar pattern, suggesting that softer or more compliant mats may extend the landing duration and reduce the rate of loading^27^. This temporal delay may be critical for lowering injury risk, especially during repeated high-impact tasks common in gymnastics training.

### Differences between gymnasts and non-gymnasts

While group differences in peak force were not statistically significant, gymnasts exhibited higher peak VGRFmax compared with the control group, which likely reflected the adoption of a ‘stiffer’ landing posture characterized by reduced joint flexion to enhance stability and meet technical requirements^28^. On the other hand, we found significant differences in kinematic parameters, with gymnasts displaying markedly different strategies, particularly in ankle and knee motion. Gymnasts exhibited consistently plantarflexion at initial contact and reduced knee flexion and knee range of motion (ROM) compared to non-gymnasts. Conversely, non-gymnasts adopted deeper, more compliant knee landings, which may reflect a natural protective mechanism in untrained individuals. These findings are in line with the study by Christoforidou et al.^29^, which highlighted that gymnasts exhibit greater plantarflexion at initial contact and reduced knee flexion and joint range of motion during landing, reflecting a joint-stiffening strategy developed through training. Whereas the non-gymnasts used greater knee ROM, possibly relying more on joint flexion for force absorption.

These differences were further highlighted in the significant group effects for joint angles and ROM, particularly at the knee, as well as in mat × group interactions for time to peak and angular velocity. Gymnasts exhibit distinct joint mechanics compared to controls, which persist across surface types. Notably, gymnasts appeared to preserve their technique across mat types, whereas non-gymnasts showed greater adaptation, particularly on softer surfaces. This may reflect the gymnasts’ familiarity with managing impact under variable surface conditions, a skill less developed in the general population. Similarly, ωKneemax demonstrated the lowest values for TYPE 2, 3, and FIG, highlighting differences in knee stabilization across landing surfaces. The absence of significant differences in ωAnklemax suggests that ankle angular velocity may not be as sensitive to surface compliance variations as knee parameters, potentially due to the prioritization of knee joint mechanics in shock absorption strategies during landings^30^.

### Implications for mat testing and development

Our findings reinforce the need for rigorous biomechanical analysis in the evaluation and development of landing surfaces. Current FIG testing protocols emphasize objective mechanical thresholds, but may benefit from including human movement data to better capture functional performance and interindividual variability. Differences in mat stiffness and material composition were clearly reflected in kinetic and kinematic metrics, demonstrating that human-based biomechanical testing can help differentiate mat types that otherwise meet similar mechanical standards.

Furthermore, the observed intergroup differences emphasize that surface evaluation should consider user populations. Mats designed exclusively around elite-level biomechanics may not serve recreational or developing athletes with different motor control strategies and joint tolerances, which is particularly important given that such landing mats are also widely used in a physical education environment. Future research should expand on these findings by incorporating longitudinal injury surveillance and musculoskeletal modelling to refine the development of landing mats that enhance safety while maintaining performance standards.

### Limitations

One of the limitations of this study is that the standardized drop landing protocol, while ensuring experimental control and comparability across conditions, may not fully reflect the complexity and variability of dynamic landings performed during actual gymnastics routines. Consequently, the findings should be interpreted with caution when extrapolating to sport-specific tasks such as apparatus dismounts, which involve different approach velocities, movement strategies, and fatigue states. It is also important to note that the current findings are specific to young female participants, and caution should be taken when extrapolating these results to male or elite-level athletes. Future research should investigate how sex and skill level may influence landing biomechanics and interactions with different mat structures, including landings following more complex skills (e.g., somersaults).

## Conclusion

This study provides evidence that both mat construction and individual training background significantly influence landing biomechanics. Mat design—including the number and type of layers—affects not only peak forces but also movement strategies, with softer mid-layers and longer loading durations associated with more favourable force profiles. Differences between gymnasts and untrained individuals emphasize the need for surface optimization tailored to different skill levels. Gymnasts exhibit distinct joint mechanics compared to controls, which persist across surface types. Future standards for mat testing and development should consider integrating biomechanical analysis across user populations to better inform safe and effective surface design.

## Data Availability

The author confirms that all data generated or analysed during this study are included in this published article. Furthermore, the raw data are stored in the Zenodo repository (10.5281/zenodo.16881141), and any further information for the reanalysis of data reported in this paper will be made available from the lead contact (pavel.brtva@osu.cz) upon request.
